# Quality of Life Analysis in SRC + HIPEC Patients: Single-Center Experience

**DOI:** 10.3390/jcm15103721

**Published:** 2026-05-12

**Authors:** Serkan Ademoğlu, İsa Caner Aydın, Ahmet Orhan Sunar, Uğur Duman, Orhan Uzun, Selçuk Gülmez, Mustafa Duman, Erdal Polat

**Affiliations:** 1 Gastroenterologic Surgery Department, Gaziantep City Hospital, Ministry of Health, 27470 Gaziantep, Türkiye; serkan.ademoglu@saglik.gov.tr; 2 Gastroenterologic Surgery Department, Zonguldak Atatürk State Hospital, Ministry of Health, 67030 Zonguldak, Türkiye; 3 Gastroenterologic Surgery Department, Koşuyolu Yüksek İhtisas Training and Research Hospital, University of Health Sciences, 34865 İstanbul, Türkiye; 4 General Surgery Department, Bursa Yüksek İhtisas Training and Research Hospital, University of Health Sciences, 16310 Bursa, Türkiye; 5 Gastroenterologic Surgery Department, Medicalpark Pendik Hospital, 34899 İstanbul, Türkiye

**Keywords:** cytoreductive surgery, HIPEC, quality of life, postoperative recovery, patient-reported outcomes, peritoneal surface malignancies

## Abstract

**Objective:** Cytoreductive surgery (CRS) combined with hyperthermic intraperitoneal chemotherapy (HIPEC) is an aggressive locoregional treatment for selected patients with peritoneal surface malignancies. Although its oncological role has been widely discussed, longitudinal data focusing on postoperative quality-of-life (QoL) trajectories remain limited. This study aimed to describe longitudinal QoL trajectories during the first two years after CRS/HIPEC and to provide exploratory descriptive comparisons according to selected clinical characteristics. **Methods:** This retrospective single-center cohort study included 144 consecutive adult patients who underwent CRS/HIPEC between January 2018 and July 2022. Patients were evaluated preoperatively and postoperatively at day 14 and at 1, 3, 6, 12, 18, and 24 months. QoL was assessed during routine follow-up primarily using the EORTC QLQ-C30 questionnaire; the EORTC QLQ-CR29 was also administered in institutional practice as a supplementary instrument in selected settings. Repeated QLQ-C30 measurements were analyzed descriptively using the Friedman test with post hoc Nemenyi comparisons. **Results:** Questionnaire completion rates were 100% at baseline, postoperative day 14, and month 1; 96.5% at months 3 and 6; 91.0% at month 12; 81.9% at month 18; and 75.7% at month 24. Longitudinal analyses demonstrated significant time effects across multiple QoL domains, including global health status, physical functioning, emotional functioning, role functioning, cognitive functioning, social functioning, pain, fatigue, and diarrhea. The most pronounced deterioration was observed in the early postoperative period, particularly at postoperative day 14. Thereafter, several domains improved gradually; however, recovery was domain-specific and did not consistently return to preoperative levels during follow-up. In exploratory descriptive analyses, patients with major postoperative complications showed more pronounced early impairment in global health status, physical functioning, and social functioning, together with greater pain and fatigue burden, particularly at postoperative day 14 and month 1. Exploratory subgroup comparisons also suggested heterogeneity in recovery patterns according to primary tumor origin. Later follow-up findings should be interpreted cautiously in view of attrition over time and the absence of adjusted longitudinal modeling. **Conclusions:** Quality of life declines substantially during the early postoperative period after CRS/HIPEC, followed by gradual but incomplete recovery over time. This recovery pattern is non-linear and varies across domains. Exploratory descriptive findings suggested that early postoperative QoL impairment may be greater in patients with major complications, but these subgroup patterns require confirmation in prospectively designed studies using adjusted longitudinal models. Longitudinal QoL assessment may provide clinically meaningful insight into postoperative recovery after CRS/HIPEC.

## 1. Introduction

Peritoneal carcinomatosis (PC) represents an advanced pattern of tumor dissemination arising from colorectal, gastric, ovarian, mesothelioma, and primary peritoneal malignancies [1]. Peritoneal spread is generally associated with poor prognosis and reduced survival compared with patients without peritoneal involvement. In selected patients, cytoreductive surgery (CRS) combined with hyperthermic intraperitoneal chemotherapy (HIPEC) has been shown to improve long-term oncological outcomes and survival [2]. However, this aggressive multimodal treatment is accompanied by substantial perioperative stress and may lead to postoperative complications such as anastomotic leakage, hemorrhage, peritonitis, ileus, wound infection, pancreatitis, intestinal fistula, urinary tract infection, sepsis, and hematologic toxicity [3,4]. Although such morbidity is often considered acceptable in appropriately selected patients, postoperative complications may adversely affect recovery and may delay the initiation of adjuvant treatment. In this context, optimized perioperative care, including enhanced recovery after surgery (ERAS) pathways, may contribute to reduced postoperative morbidity and improved functional recovery after major oncologic procedures [5].

Patient selection for CRS/HIPEC is primarily based on clinical fitness, tumor biology, disease burden, response to systemic treatment, and the presence or absence of extra-abdominal disease [6]. Although baseline performance status and daily functional capacity are routinely considered during decision-making, baseline quality of life (QoL) is not usually incorporated as a central selection parameter. Nevertheless, growing evidence suggests that QoL is not merely a descriptive endpoint, but may also be related to treatment tolerance, postoperative recovery, and survival-related outcomes [7,8].

Previous studies evaluating QoL after CRS/HIPEC have consistently shown a marked deterioration during the early postoperative period, followed by gradual recovery over time, with several domains approaching baseline levels within 6 to 12 months in some cohorts [9,10,11]. However, the available literature remains limited regarding long-term longitudinal QoL trajectories in clinically heterogeneous CRS/HIPEC populations. In addition, no validated CRS/HIPEC-specific QoL instrument currently exists, and available assessments are largely based on general cancer-related instruments or tumor-specific modules adapted from broader oncologic populations [12,13]. Therefore, the present study aimed to describe longitudinal quality-of-life trajectories during the first two years after CRS/HIPEC in a real-world, clinically heterogeneous single-center cohort. As secondary exploratory analyses, we also evaluated whether postoperative QoL patterns appeared to vary according to selected clinical characteristics, particularly primary tumor origin and postoperative complication severity.

## 2. Materials and Methods

This retrospective single-center cohort study was conducted at a tertiary referral center with dedicated experience in cytoreductive surgery (CRS) and hyperthermic intraperitoneal chemotherapy (HIPEC). Consecutive adult patients (aged ≥18 years) who underwent CRS/HIPEC at our institution between January 2018 and July 2022 were screened for eligibility. All consecutive patients who met the inclusion criteria were included, without selective omission. QoL data were obtained from questionnaires administered at predefined perioperative and follow-up visits as part of routine clinical care and retrospectively analyzed for the present study. Patients with available baseline and postoperative questionnaire data were included in the longitudinal descriptive analyses according to data availability at each scheduled time point.

Demographic data, comorbidities, primary tumor origin, perioperative characteristics, and postoperative outcomes were retrospectively obtained from institutional medical records. In addition to the principal longitudinal QoL assessments, several clinical and perioperative variables were recorded to characterize the cohort and provide contextual information regarding postoperative recovery, including postoperative complications, length of hospital stay, intensive care unit stay, 30-day readmission, recurrence, completeness of cytoreduction, ostomy formation, transfusion requirement, and exposure to systemic chemotherapy. However, these variables were not incorporated into a formal adjusted longitudinal model of QoL trajectories in the present analysis.

This study was conducted in accordance with the Declaration of Helsinki and was approved by the Institutional Review Board of İstanbul Kartal Koşuyolu Research and Training Hospital (approval number: 2022/15/631; 11 October 2022). Because this study was based exclusively on routinely collected clinical follow-up data, no additional study-specific intervention or assessment was performed.

QoL analyses were based on evaluable questionnaires available at each scheduled assessment time point. No formal imputation strategy was applied for missing longitudinal QoL data. Because questionnaire completion during follow-up depended on continued survival, clinical status, and attendance at routine visits, later assessments may disproportionately reflect patients who remained alive, clinically stable, and available for follow-up. Questionnaires were collected during inpatient or scheduled outpatient follow-up encounters; no separate telephone- or electronically administered QoL collection protocol was used in the present study.

Quality-of-life assessment in routine follow-up was performed using the European Organisation for Research and Treatment of Cancer Core Quality of Life Questionnaire (EORTC QLQ-C30). In our institutional practice, the EORTC Colorectal Quality of Life Questionnaire (QLQ-CR29) was also administered as a supplementary instrument in selected follow-up settings, largely reflecting the historical predominance of colorectal and colorectal-like peritoneal surface malignancies in CRS/HIPEC practice. However, because the present cohort included multiple non-colorectal primary tumor groups and no CRS/HIPEC-specific validated QoL instrument is currently available, the principal longitudinal analyses presented in this study were based on EORTC QLQ-C30 domain scores [12,13]. Questionnaire responses were scored according to the EORTC scoring manual and linearly transformed into standardized 0–100 scores. For global health status and functional scales, higher scores indicate better functioning or better overall quality of life, whereas for symptom scales, higher scores indicate greater symptom burden. Patients completed the questionnaires preoperatively and postoperatively at day 14 and at 1, 3, 6, 12, 18, and 24 months after surgery as part of routine clinical follow-up. Questionnaires were administered during hospitalization and scheduled outpatient visits after discharge, and were completed independently or with assistance from a member of the clinical team when necessary. The principal longitudinal analyses presented in this manuscript were based on QLQ-C30 domain scores transformed to the standardized 0–100 metric.

Patients who had received preoperative systemic chemotherapy underwent CRS/HIPEC only after an interval of at least 4 weeks from the last chemotherapy cycle. Postoperative systemic chemotherapy, when indicated, was generally resumed approximately 1 month after surgery according to clinical recovery and the oncological treatment plan.

### Statistical Analysis

Continuous variables were summarized as mean ± standard deviation or median (minimum–maximum/interquartile range), as appropriate, and categorical variables as number (percentage). Distributional normality was assessed using the Shapiro–Wilk test. Because repeated QoL measurements were not normally distributed, longitudinal within-cohort comparisons across time points were performed using the Friedman test. Post hoc pairwise comparisons were conducted using the Nemenyi procedure in Python. Descriptive analyses were performed in IBM SPSS Statistics for Windows, version 22.0 (IBM Corp., Armonk, NY, USA). A two-sided *p* value < 0.05 was considered statistically significant.

## 3. Results

A total of 144 consecutive patients were included in this study. The mean age was 54.6 ± 11.3 years (range, 24–73 years), and 77 patients (53.5%) were female. Most patients were classified as ASA III–IV (94.4%). The most common primary tumor origin was colorectal cancer (52.8%), followed by pseudomyxoma peritonei (15.3%), gastric cancer (13.2%), ovarian cancer (12.5%), and malignant mesothelioma (6.2%). The study cohort was clinically heterogeneous with respect to primary tumor origin, including colorectal, gastric, ovarian, pseudomyxoma peritonei, and malignant mesothelioma cases. Accordingly, pooled longitudinal QoL findings should be interpreted as descriptive whole-cohort patterns rather than tumor-specific recovery estimates. Questionnaire completion rates were 100% at baseline, postoperative day 14, and postoperative month 1; 96.5% at postoperative month 3 and month 6; 91.0% at postoperative month 12; 81.9% at postoperative month 18; and 75.7% at postoperative month 24 (Table 1).

To assess the availability of evaluable quality-of-life questionnaires across scheduled follow-up time points, the analyses were based on available questionnaires at each assessment. Because this study was retrospective and based on routine follow-up, missing questionnaires at later time points may reflect nonattendance, clinical deterioration, loss to follow-up, or death.

**Figure 1:**
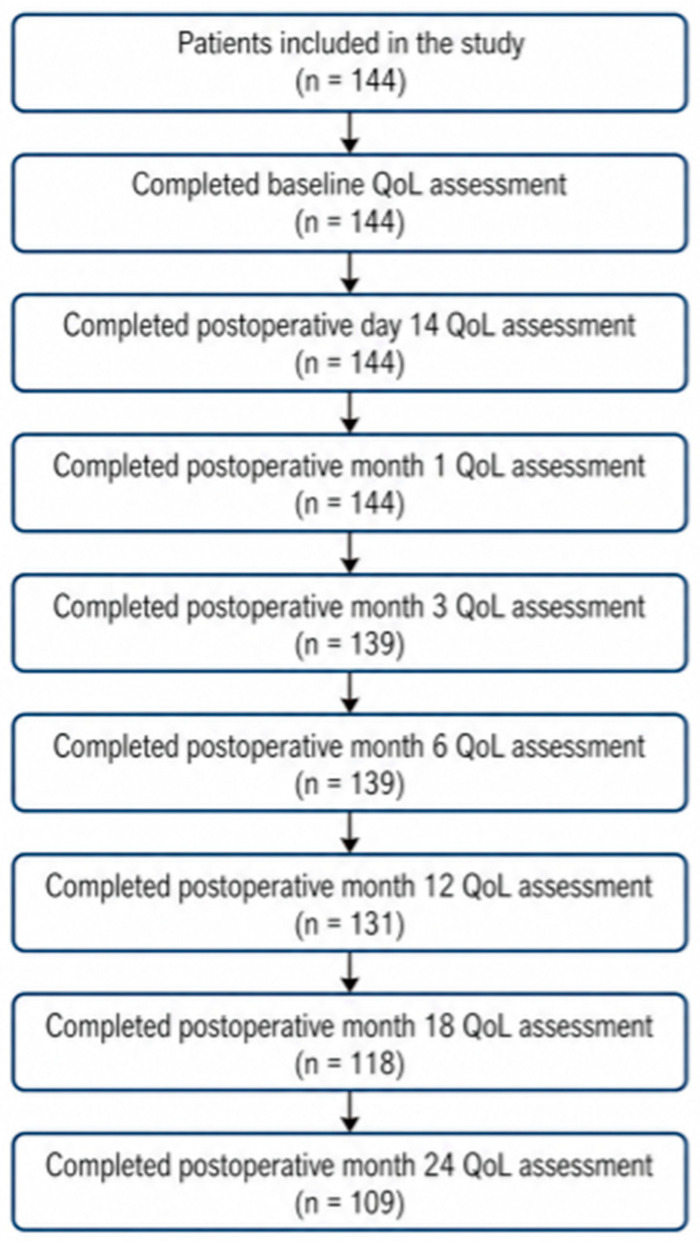
Availability of evaluable longitudinal quality-of-life assessments during follow-up.

Follow-up completeness declined progressively over time, with evaluable questionnaires available in 139 of 144 patients (96.5%) at postoperative months 3 and 6, 131 of 144 patients (91.0%) at month 12, 118 of 144 patients (81.9%) at month 18, and 109 of 144 patients (75.7%) at month 24. Therefore, longitudinal findings at later follow-up time points should be interpreted with caution, as they may partly reflect attrition and survivorship-related selection rather than unbiased cohort-wide recovery alone (Figure 1).

Previous systemic chemotherapy had been administered to 97 patients (67.4%). Complete cytoreduction (CC-0) was achieved in 134 patients (93.1%). Ileostomy and colostomy were performed in 25 (17.6%) and 11 (7.6%) patients, respectively. The mean operative time was 7.59 ± 3.14 h, the mean intensive care unit stay was 1.88 ± 2.72 days, and the mean length of hospital stay was 13.39 ± 10.59 days; the median hospital stay was 10 days (range, 4–77 days). The mean Peritoneal Cancer Index (PCI) was 4.92 ± 3.40. Perioperative and surgical characteristics are summarized in Table 2.

These perioperative and oncologic variables are presented to characterize the clinical context of the cohort; however, they were not formally modeled as independent predictors of longitudinal QoL trajectories in the present analysis.

Longitudinal analyses of standardized EORTC QLQ-C30 scores demonstrated significant time effects across multiple QoL domains, including global health status, physical functioning, emotional functioning, role functioning, cognitive functioning, social functioning, pain, fatigue, and diarrhea. Because the present cohort included multiple non-colorectal primary tumor groups, the principal longitudinal analyses were restricted to EORTC QLQ-C30 domains, which were considered the most broadly applicable patient-reported outcome measures across the whole study population. In general, global health status and several functional domains worsened during the early postoperative period, particularly at postoperative day 14, whereas symptom burden increased for pain and fatigue. Although gradual improvement was observed during subsequent follow-up, recovery remained domain-specific and was not uniformly complete by postoperative month 24. Longitudinal analyses using standardized 0–100 EORTC QLQ-C30 scores demonstrated statistically significant time effects across multiple quality-of-life domains, including global health status, physical functioning, emotional functioning, role functioning, cognitive functioning, social functioning, pain, fatigue, and diarrhea. The longitudinal analyses presented in Table 3 were based on EORTC QLQ-C30 domain scores transformed to the standardized 0–100 metric.

Post hoc pairwise comparisons confirmed significant differences between the preoperative assessment and postoperative day 14 in several clinically relevant domains. Global health status, physical functioning, social functioning, pain, and fatigue showed marked early postoperative worsening, followed by significant improvement over subsequent follow-up. Improvement between postoperative day 14 and postoperative month 12 was significant in several of these domains, whereas recovery by month 24 remained incomplete for selected measures. By contrast, emotional and cognitive functioning showed relatively limited fluctuation over time, and dyspnea did not demonstrate a consistent clinically meaningful longitudinal change. However, the absolute magnitude of several observed score differences was limited, and these statistically significant changes should not automatically be interpreted as uniformly clinically meaningful. For consistency of interpretation, postoperative worsening in global health status and functional domains was expressed as a positive difference by calculating baseline score minus postoperative score, whereas symptom worsening was expressed as a positive difference by calculating postoperative score minus baseline score.

**Table 4:** Post hoc summary of selected clinically relevant pairwise comparisons in QoL domains.

PRO	Preop vs. Day 14	Day 14 vs. Month 12	Day 14 vs. Month 24
General Health	<0.001 *	<0.001 *	<0.001 *
Physical Function	<0.001 *	<0.001 *	0.011 *
Social Function	<0.001 *	<0.001 *	<0.001 *
Emotional Function	0.025 *	0.439	0.489
Cognitive Function	0.347	0.638	0.464
Pain	<0.001 *	<0.001 *	0.011 *
Fatigue	<0.001 *	<0.001 *	0.015 *
Dyspnea	0.412	0.587	0.601

Note: *p* values are derived from post hoc pairwise comparisons using the Nemenyi test following significant Friedman test results. * *p* < 0.05 indicates statistical significance.

**Figure 2:**
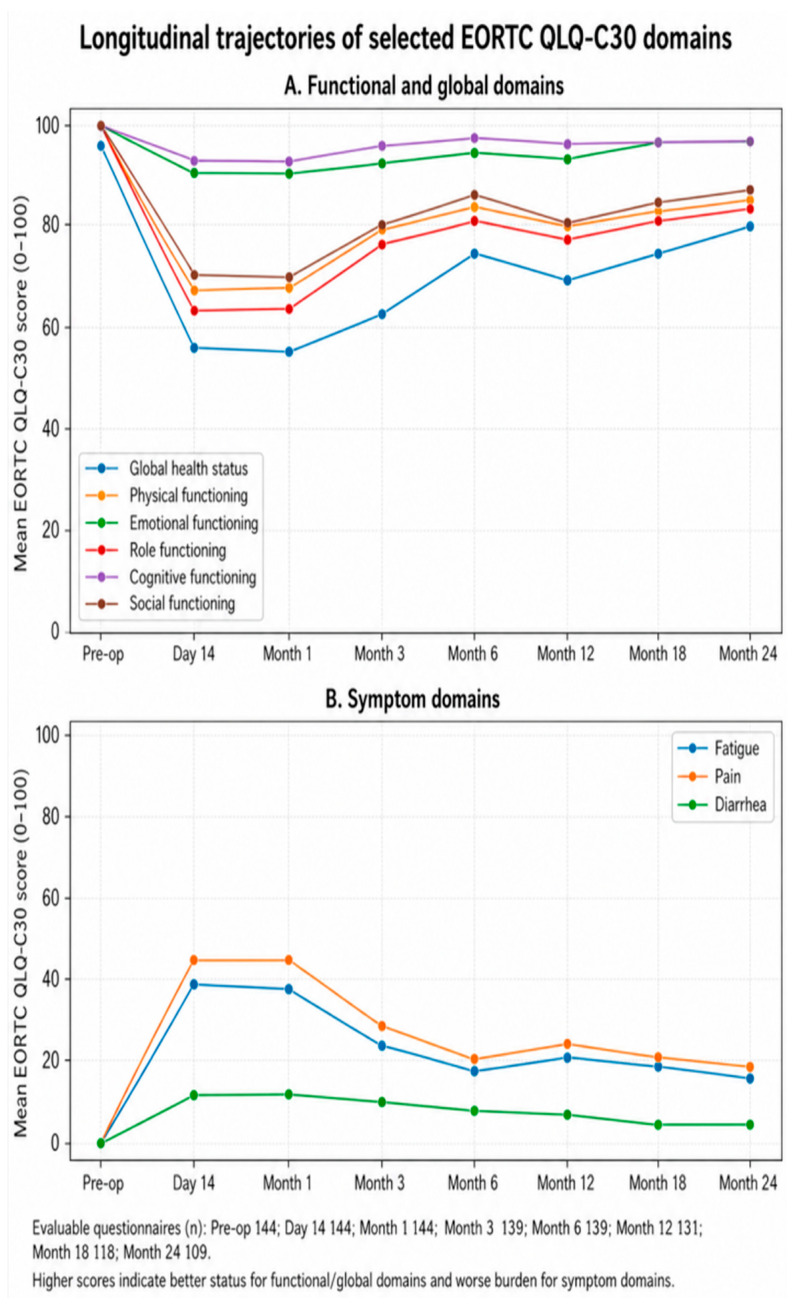
Longitudinal trajectories of selected EORTC QLQ-C30 domains. (**A**) Functional and global domains: global health status, physical functioning, emotional functioning, role functioning, cognitive functioning, and social functioning. (**B**) Symptom domains: fatigue, pain, and diarrhea. Note: The figure is intended as a visual summary of the descriptive longitudinal QoL patterns and does not replace the formal statistical comparisons presented in Table 3 and Table 4. Data are presented as mean EORTC QLQ-C30 scores across the scheduled assessment time points: preoperative, postoperative day 14, and postoperative months 1, 3, 6, 12, 18, and 24. Higher scores indicate better status for functional/global domains and greater symptom burden for symptom domains. Evaluable questionnaires were available for 144 patients preoperatively, 144 at postoperative day 14, 144 at month 1, 139 at month 3, 139 at month 6, 131 at month 12, 118 at month 18, and 109 at month 24.

To facilitate visual interpretation of these repeated-measures findings, the longitudinal trajectories of the selected QLQ-C30 functional/global and symptom domains are presented in Figure 2.

Postoperative complications were classified according to the Clavien–Dindo system. Minor complications (grade I–II) occurred in 106 patients (73.6%), whereas major complications (grade III–IV) were observed in 38 patients (26.4%) (Table 5).

Minor complications were defined as Clavien–Dindo grade I–II, and major complications as grade III–IV.

Exploratory descriptive comparisons according to complication severity suggested that patients with major postoperative complications experienced greater early deterioration in several clinically relevant QoL domains than those with minor complications. In particular, lower global health status, physical functioning, and social functioning scores, together with higher pain and fatigue burden, were more evident during the early postoperative period, especially at postoperative day 14 and postoperative month 1. However, because these observations were based on descriptive subgroup comparisons without adjusted between-group longitudinal modeling, they should be interpreted cautiously and regarded as hypothesis-generating rather than confirmatory (Table 6).

Exploratory descriptive comparisons suggested that postoperative QoL recovery patterns were not entirely uniform across primary tumor groups. However, because several tumor-specific subgroups were limited in size and no adjusted longitudinal modeling was performed, these observations should be interpreted cautiously and should not be considered definitive comparative evidence (Table 7).

Exploratory descriptive comparisons according to ostomy status suggested that postoperative recovery patterns were not entirely uniform between patients with and without an ostomy. In particular, patients with any ostomy appeared to have less favorable recovery in selected domains, especially physical functioning, social functioning, pain, and fatigue at later follow-up time points. However, because these observations were based on descriptive subgroup summaries without adjusted longitudinal modeling, they should be interpreted cautiously and regarded as hypothesis-generating rather than confirmatory (Table 8).

**Figure 3:**
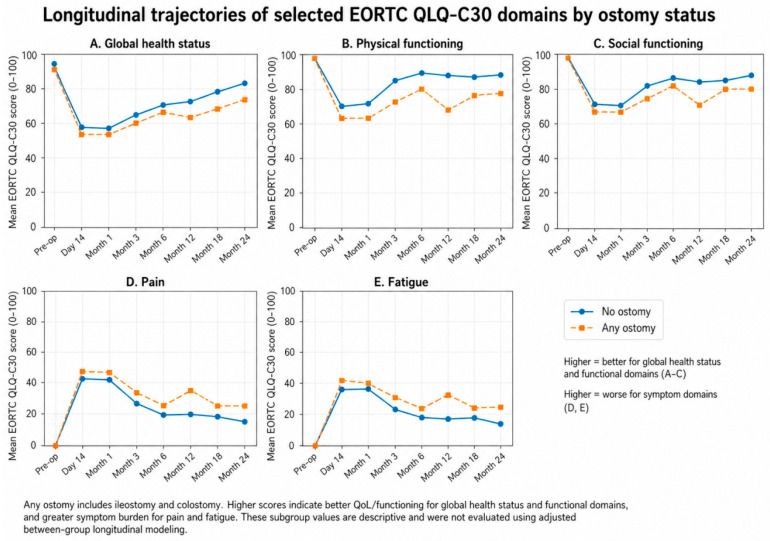
Exploratory longitudinal trajectories of selected EORTC QLQ-C30 domains according to ostomy status. Mean scores are shown for patients without an ostomy and those with any ostomy across follow-up. Any ostomy included ileostomy and colostomy. Higher scores indicate better status for global health/functional domains and worse symptom burden for symptom domains. These subgroup trajectories are presented descriptively and were not evaluated using adjusted between-group longitudinal modeling.

The exploratory longitudinal trajectories of selected QLQ-C30 domains according to ostomy status are further illustrated in Figure 3, which visually summarizes differences in selected functional and symptom domains between patients with and without an ostomy during follow-up.

## 4. Discussion

In the present study, we evaluated longitudinal quality-of-life (QoL) trajectories in patients undergoing cytoreductive surgery (CRS) followed by hyperthermic intraperitoneal chemotherapy (HIPEC). The principal findings can be summarized as follows: First, QoL deteriorated substantially during the early postoperative period, particularly at postoperative day 14, across several functional and symptom-related domains. Second, most domains showed gradual improvement over time, although recovery was not uniform and complete return to preoperative baseline was not consistently achieved within the observation period. Third, exploratory descriptive comparisons suggested that patients with major postoperative complications may experience more pronounced early impairment across selected QoL domains. Finally, exploratory subgroup comparisons suggested that postoperative recovery patterns may vary according to tumor origin, further underscoring the clinical heterogeneity of CRS/HIPEC populations.

Previous studies have consistently shown that CRS/HIPEC is associated with considerable short-term postoperative burden despite its potential oncological benefit [9,14,15]. This interpretation is also aligned with contemporary systematic and narrative reviews indicating that CRS/HIPEC remains a highly selected treatment strategy whose potential clinical benefit must be balanced against substantial perioperative burden and the need for meticulous perioperative management [16,17]. In line with these reports, our findings demonstrated marked early worsening in patient-reported outcomes, especially in general health status, physical functioning, social functioning, pain, and fatigue. This early decline is clinically plausible in the context of CRS/HIPEC, which is a major procedure often involving prolonged operative time, multivisceral resection, ostomy formation, postoperative pain, and delayed functional recovery. Exploratory descriptive analysis according to ostomy status also suggested that patients with an ostomy may experience less favorable recovery in selected QoL domains, particularly physical functioning, social functioning, pain, and fatigue. This observation is clinically plausible given the potential physical and psychosocial burden associated with ostomy care after extensive abdominal surgery. However, these findings should be interpreted cautiously because no adjusted longitudinal modeling was performed and the ostomy subgroup remained clinically heterogeneous, particularly when ileostomy and colostomy were considered together. In addition, broader oncology literature suggests that recovery after major cancer surgery may be modifiable through structured supportive interventions, whereas severe postoperative events such as anastomotic leakage may further compromise short-term recovery and patient well-being [18,19]. The subsequent improvement observed during follow-up is also consistent with prior prospective QoL studies reporting partial recovery within months after surgery [9,14,15]. However, our results suggest that recovery is domain-specific rather than uniform, and that overall patient-perceived well-being may recover more slowly than isolated functional parameters. An additional point that should be considered is the distinction between statistical significance and clinical relevance. Although several QLQ-C30 domains showed statistically significant changes over time, the absolute magnitude of many observed differences was relatively small. In the context of EORTC QLQ-C30 interpretation, such changes may not uniformly exceed commonly used thresholds for minimal clinically important difference (MCID). Therefore, the early postoperative deterioration identified in the present study should be interpreted as statistically detectable but clinically modest in magnitude for several domains, rather than as evidence of uniformly large QoL impairment across the cohort.

Another methodological consideration relates to instrument selection and score interpretation. Although the QLQ-CR29 was used in our institutional follow-up practice, the present study relied primarily on QLQ-C30 domains for whole-cohort longitudinal analysis because the study population was clinically heterogeneous and included several non-colorectal malignancies. In this setting, QLQ-C30 offered a more broadly applicable framework for repeated assessment of global health, functioning, and symptom burden across disease groups. To avoid misinterpretation of score direction, longitudinal QoL changes were interpreted according to established EORTC scale conventions, with separate consideration of functional and symptom domains and explicit clarification of subtraction direction in Table 3. Nevertheless, the absence of a CRS/HIPEC-specific validated QoL instrument remains an important limitation in this field.

The clinical heterogeneity of the cohort should be carefully considered when interpreting the present findings. Patients undergoing CRS/HIPEC do not constitute a single oncological entity, and our cohort included colorectal cancer, gastric cancer, ovarian cancer, pseudomyxoma peritonei, and malignant mesothelioma. These tumor types differ substantially with respect to tumor biology, perioperative treatment strategies, recurrence patterns, symptom burden, and expected survivorship. Accordingly, the pooled longitudinal QoL profile reported here should not be interpreted as a disease-specific trajectory, but rather as a descriptive overview of postoperative recovery in a real-world mixed CRS/HIPEC cohort. Exploratory subgroup comparisons suggested some variation in recovery patterns according to primary tumor origin; however, these findings should be regarded as hypothesis-generating only, because the present study was not designed or powered to provide robust tumor-specific comparisons. This issue may be particularly relevant in gastric cancer, in which the therapeutic role and postoperative course of CRS/HIPEC differ from those observed in other primary tumor settings [20]. Future prospective studies with tumor-stratified analyses will be necessary to determine whether QoL trajectories after CRS/HIPEC differ meaningfully across disease entities.

Another important consideration is the favorable selection profile of the present cohort. The mean PCI was relatively low, the rate of complete cytoreduction (CC-0) was high, and perioperative recovery indicators such as ICU stay and length of hospitalization were generally compatible with a carefully selected operable population. These features likely reflect institutional selection practices favoring patients with lower disease burden and greater procedural suitability for CRS/HIPEC. Accordingly, the present findings should be interpreted with caution when considering external validity, because the reported QoL trajectories may not be directly generalizable to centers treating patients with higher PCI scores, less complete cytoreduction, or a heavier overall burden of disease.

Later postoperative QoL patterns should not be interpreted as reflecting surgical recovery alone. In a mixed oncological cohort such as this, recurrence, postoperative systemic treatment, and the broader disease course are clinically plausible contributors to variation in later patient-reported outcomes. However, these factors were not formally modeled in the present analysis, and their independent effects should therefore not be inferred directly from the observed longitudinal patterns. Accordingly, later QoL findings should be interpreted cautiously within the broader oncological framework rather than as isolated consequences of CRS/HIPEC alone [21].

Our exploratory analysis according to complication severity suggested that patients with major postoperative complications experienced more pronounced early deterioration in global health, physical functioning, social functioning, pain, and fatigue. These descriptive differences were most apparent during the early postoperative phase, particularly at postoperative day 14 and month 1, whereas they appeared less marked by month 12; however, this later convergence should be interpreted cautiously, as it may reflect recovery, variability related to smaller follow-up subsets, or survivorship-related selection. This study was not designed to formally model between-group longitudinal differences over time, and postoperative morbidity was summarized primarily as a major-versus-minor severity grouping without detailed analysis of complication subtype, timing, cumulative burden, or the specific contribution of reoperation. Therefore, the present data support only the cautious interpretation that more severe postoperative morbidity may be associated with worse early recovery patterns, but do not establish an independent effect of complications on longitudinal QoL trajectories.

Similarly, perioperative variables such as ICU stay, length of hospital stay, transfusion requirement, and completeness of cytoreduction are clinically relevant markers of treatment intensity and recovery burden, but they were not examined within an adjusted longitudinal QoL framework in the present analysis. As a result, no direct inference can be made regarding their independent contribution to postoperative QoL trajectories.

The longitudinal QoL findings also offer an important practical message. Recovery after CRS/HIPEC should not be understood as a single transition from postoperative decline to full restoration. Rather, recovery is dynamic, multidimensional, and domain-specific. While several symptom and function domains improved substantially after the early postoperative period, global health status remained below baseline general health status remained below baseline, and some domains showed incomplete or fluctuating recovery over time. In addition, the extent of surgery itself may contribute to late adverse effects and lingering impairment in selected domains, which may partly explain why full normalization was not consistently observed during follow-up [22]. A recent large prospective cohort further emphasized that postoperative QoL changes after HIPEC-based treatment should be interpreted not only statistically, but also in terms of clinically meaningful change over time, including the concept of minimal clinically important difference [23]. This is particularly relevant in the present cohort, in which several baseline functional scores were relatively high, raising the possibility that ceiling effects may have limited the apparent magnitude of postoperative recovery in selected domains. Contemporary cohort data likewise support the view that outcomes after CRS/HIPEC depend heavily on patient selection, perioperative course, and longitudinal follow-up, underscoring the importance of interpreting QoL trajectories within a broader clinical framework [24]. From a clinical standpoint, patient counseling before CRS/HIPEC should include not only discussion of morbidity and oncological expectations, but also realistic information regarding the expected time course and variability in postoperative functional recovery. Another point that should be considered when interpreting the present findings is the distribution of baseline QoL scores. In several functional domains, preoperative values were close to the favorable end of the scale, whereas symptom scores were low in multiple domains. This pattern likely reflects the fact that CRS/HIPEC is generally offered to a relatively selected operable population after detailed preoperative assessment and optimization. As a result, some degree of ceiling effect may have been present at baseline, which could magnify the apparent extent of early postoperative deterioration and influence the visual impression of later recovery.

The present study has several strengths. This study was conducted at a tertiary referral center with a dedicated multidisciplinary CRS/HIPEC program, ensuring procedural consistency in surgical technique, intraperitoneal chemotherapy delivery, and perioperative management across the entire cohort. Longitudinal follow-up extended to 24 months postoperatively, which is notably longer than many comparable single-center series and allows for the characterization of both early deterioration and late recovery trajectories across multiple QoL domains. Furthermore, the initial questionnaire completion rate was 100% at baseline and remained 100% through the first postoperative month, reflecting a structured and protocol-driven data collection approach that minimizes early attrition and ensures the reliability of the baseline and early postoperative QoL estimates. In addition, recent overviews of CRS/HIPEC practice continue to emphasize the importance of careful patient selection, multidisciplinary treatment planning, and structured follow-up, all of which provide an appropriate context for interpreting the present findings [25]. Several limitations of this study should be acknowledged. First, although repeated QoL assessments were available over a 24-month period, the present analyses were based on a descriptive nonparametric repeated-measures approach using the Friedman and Nemenyi tests rather than an adjusted mixed-effects longitudinal model. Accordingly, the analytical framework did not account for patient-specific longitudinal trajectories, did not permit formal covariate adjustment, and did not fully address incomplete repeated measurements or potentially informative missingness. This approach was selected in view of the non-normal distribution of repeated QoL measurements confirmed by the Shapiro–Wilk test and the retrospective single-center design, which precluded the a priori specification of a formal covariate-adjusted model. As a result, the independent contributions of clinically relevant variables—including age, performance status, peritoneal cancer index, completeness of cytoreduction, complication grade, primary tumor origin, and postoperative systemic treatment—to longitudinal QoL trajectories could not be formally evaluated within the present analytical framework. Therefore, the present findings should be interpreted primarily as descriptive longitudinal observations rather than as evidence of independently validated determinants of postoperative QoL recovery. Second, questionnaire completion declined during follow-up, reaching 75.7% at 24 months, which introduces the possibility of attrition bias, survivorship bias, and informative missingness. In particular, patients available for later QoL assessment may have represented a clinically fitter subgroup, potentially leading to overestimation of apparent long-term recovery. Clinically, such patients are more likely to have achieved complete cytoreduction (CC-0), to be free of disease recurrence at later follow-up time points, and to have completed or not required adjuvant systemic therapy—all of which are factors associated with more favorable postoperative trajectories and continued engagement with structured follow-up programs. Third, because this study was retrospective and exploratory in nature, subgroup patterns according to complication severity or tumor origin should be interpreted as hypothesis-generating rather than confirmatory. Fourth, no CRS/HIPEC-specific validated QoL instrument was available; therefore, the principal analyses relied on the broadly applicable EORTC QLQ-C30, while the EORTC QLQ-CR29 was used only as a supplementary instrument in selected follow-up settings. Fifth, although recurrence and postoperative systemic treatment may have influenced later QoL trajectories, these factors were not formally evaluated using time-dependent adjusted longitudinal models. Finally, survival outcomes were not retained as formal endpoints in the revised analysis because the present study was designed primarily to evaluate longitudinal QoL trajectories, and the biological heterogeneity of the cohort would limit the interpretability of survival-based subgroup inference. In addition, the relatively low mean PCI and high CC-0 rate suggest that the study population was favorably selected, which may limit the generalizability of the findings to higher-burden CRS/HIPEC populations. Another important limitation is the marked clinical heterogeneity of the cohort, which included several distinct primary tumor groups with different biological behavior, therapeutic pathways, and survivorship expectations. This limits the disease-specific interpretability of pooled QoL estimates and reduces the extent to which the present results can be generalized to any single tumor population. Importantly, the present study was designed primarily to describe longitudinal QoL trajectories rather than to establish independent determinants of postoperative QoL change. Therefore, observations related to complication severity, tumor origin, recurrence, and treatment-related factors should be interpreted as exploratory descriptive findings. More robust prospective studies using adjusted longitudinal models will be necessary to determine the independent contribution of these variables to postoperative QoL trajectories. In addition, although a colorectal-specific supplementary module was used in routine practice, the mixed-disease composition of the cohort limited the cross-disease interpretability of such instrument-specific data. This further supports our decision to base the principal longitudinal analyses on QLQ-C30 domains, but also underscores the need for CRS/HIPEC-specific patient-reported outcome tools. An ideal CRS/HIPEC-specific instrument would need to capture procedure-related symptom dimensions not adequately represented in generic or tumor-specific modules, including abdominal distension, intraperitoneal chemotherapy-related toxicity, stoma-related burden, and the psychosocial impact of a complex oncological intervention with uncertain long-term prognosis. In addition, several baseline functional scores were close to the favorable end of the scale and symptom scores were low in multiple domains, suggesting a possible ceiling effect related to preoperative patient selection and optimization. This may have accentuated the apparent magnitude of early postoperative decline in some domains. Consequently, the true extent of postoperative functional impairment may have been underestimated in domains where preoperative scores were already near the upper limit of the scale, and readers should account for this possibility when interpreting domain-specific recovery patterns. We also acknowledge that, although questionnaire availability across follow-up was summarized visually, this study did not include a fully structured responder-versus-nonresponder comparison between patients with more complete versus less complete QoL follow-up. As a result, the mechanisms underlying missingness could not be fully characterized, and the extent to which later responders differed systematically from nonresponders cannot be precisely determined from the present dataset. Because no formal imputation strategy or responder-versus-nonresponder analysis was performed, missing longitudinal observations could not be assumed to be random. This limits confidence in the interpretation of late postoperative QoL trends and increases the possibility that apparent long-term recovery was influenced by selective follow-up retention. Furthermore, postoperative morbidity was analyzed using a simplified major-versus-minor complication grouping, without detailed evaluation of complication subtype, onset timing, cumulative burden, or reoperation-related impact on quality of life. This limits the precision of the complication analysis and reduces the extent to which specific postoperative events can be linked to the observed QoL patterns. Moreover, several clinically relevant perioperative and oncologic variables—including recurrence, postoperative systemic treatment, ostomy formation, ICU stay, length of hospital stay, transfusion requirement, and completeness of cytoreduction—were collected descriptively but were not incorporated into an adjusted longitudinal QoL model. Therefore, their potential influence on postoperative quality-of-life patterns could not be evaluated directly in the present study.

An additional issue affecting interpretation of the later follow-up results is the possibility of survivorship bias. In a surgical oncology cohort such as this, patients who remain alive, clinically stable, and engaged in follow-up are more likely to contribute QoL data at 12, 18, and 24 months. Therefore, the apparent partial recovery observed in some domains over time may not solely represent true cohort-wide recovery, but may also reflect selective retention of healthier survivors. This consideration is particularly important in the context of recurrence, ongoing systemic treatment, and differential clinical trajectories after CRS/HIPEC. Taken together, these limitations indicate that the present findings should be interpreted primarily as a descriptive longitudinal overview rather than as evidence of independently validated determinants of postoperative QoL recovery. Future prospective studies using mixed-effects longitudinal modeling with predefined covariate adjustment and structured handling of missing data are needed to more precisely characterize QoL trajectories after CRS/HIPEC and to distinguish treatment-related recovery from survivorship-related selection.

## 5. Conclusions

Quality of life declines substantially during the early postoperative period after cytoreductive surgery and hyperthermic intraperitoneal chemotherapy, followed by gradual but incomplete recovery over time. This recovery pattern is domain-specific and non-linear. Exploratory descriptive findings suggested that patients with major postoperative complications may show greater early impairment in selected QoL domains, particularly during the first postoperative month. Later postoperative patterns should be interpreted cautiously, as the present study was not designed to formally evaluate the independent contribution of recurrence, postoperative systemic treatment, or other perioperative variables to long-term QoL recovery. Because these findings were derived from a clinically heterogeneous mixed-tumor cohort, they should be interpreted primarily as a descriptive overview of recovery after CRS/HIPEC rather than as a tumor-specific recovery model. Future prospective studies with adjusted longitudinal modeling, tumor-specific stratification, and more structured handling of missing data are needed to better define recovery patterns in this patient population.

## Figures and Tables

**Table 1:** Baseline Demographic and Clinical Characteristics.

Variable	Value
Age, mean ± SD (range)	54.6 ± 11.3 (24–73)
Female sex, n (%)	77 (53.5)
Male sex, n (%)	67 (46.5)
ASA III–IV, n (%)	136 (94.4)
Diabetes mellitus, n (%)	22 (15.3)
Hypertension, n (%)	38 (26.4)
Coronary artery disease, n (%)	6 (4.2)
Pulmonary disease, n (%)	5 (3.5)
Primary tumor origin, n (%)	
Colorectal cancer	76 (52.8)
Gastric cancer	19 (13.2)
Ovarian cancer	18 (12.5)
Pseudomyxoma peritonei	22 (15.3)
Malignant mesothelioma	9 (6.2)
Questionnaire response rate—baseline (%)	100
Questionnaire response rate—day 14 (%)	100
Questionnaire response rate—month 1 (%)	100
Questionnaire response rate—month 3 (%)	96.5
Questionnaire response rate—month 6 (%)	96.5
Questionnaire response rate—month 12 (%)	91.0
Questionnaire response rate—month 18 (%)	81.9
Questionnaire response rate—month 24 (%)	75.7

Abbreviations: SD, standard deviation; ASA, American Society of Anesthesiologists. Note: Values are presented as mean ± SD (range) or number (%), as appropriate. Questionnaire response rates were calculated according to the availability of evaluable QoL data at each scheduled follow-up time point.

**Table 2:** Surgical procedures and perioperative characteristics.

Variable	n (%) or Value
Prior systemic chemotherapy	97 (67.4)
Peritonectomy	80 (55.6)
Diaphragmatic peritonectomy	64 (44.4)
Pelvic peritonectomy	58 (40.3)
Colon resection	79 (54.9)
Splenectomy	50 (34.7)
Gastrectomy	25 (17.4)
CC-0 cytoreduction	134 (93.1)
Ileostomy	25 (17.6)
Colostomy	11 (7.6)
Cisplatin-based HIPEC	54 (37.5)
Oxaliplatin-based HIPEC	90 (62.5)
Operation time, mean ± SD (hours)	7.59 ± 3.14
ICU stay, mean ± SD (days)	1.88 ± 2.72
Length of hospital stay, mean ± SD (days)	13.39 ± 10.59
Length of hospital stay, median (range)	10 (4–77)
Peritoneal Cancer Index, mean ± SD	4.92 ± 3.40
Erythrocyte suspension transfusion, mean ± SD (units)	0.85 ± 1.25
Fresh frozen plasma transfusion, mean ± SD (units)	1.14 ± 1.67

Abbreviations: CC-0, complete cytoreduction (no visible residual disease); HIPEC, hyperthermic intraperitoneal chemotherapy; ICU, intensive care unit; PCI, Peritoneal Cancer Index; SD, standard deviation.

**Table 3:** Longitudinal changes in standardized 0–100 EORTC QLQ-C30 scores relative to preoperative baseline, separated by functional/global and symptom domains.

Domain	Day 14	Month 1	Month 3	Month 6	Month 12	Month 18	Month 24	Friedman *p*
Functional and global domains								
Global health status	−4.67	−4.65	−3.75	−1.91	−2.54	−2.40	−2.22	<0.001 *
Physical functioning	+3.91	+3.63	+1.74	+0.88	+1.38	+1.92	+2.24	<0.001 *
Emotional functioning	+0.97	+0.97	+0.76	+0.61	+0.42	+0.26	+0.24	<0.001 *
Role functioning	+1.75	+1.64	+0.74	+0.44	+0.62	+0.83	+0.99	<0.001 *
Cognitive functioning	+0.18	+0.19	+0.16	+0.13	+0.08	+0.16	+0.14	0.010 *
Social functioning	+1.26	+1.21	+0.65	+0.31	+0.52	+0.74	+0.85	<0.001 *
Symptom domains								
Fatigue	+2.92	+2.73	+1.38	+0.82	+0.99	+1.35	+1.55	<0.001 *
Pain	+2.46	+2.36	+1.21	+0.62	+0.79	+0.96	+1.09	<0.001 *
Diarrhea	+0.40	+0.41	+0.33	+0.26	+0.21	+0.13	+0.12	0.001 *

Note: EORTC QLQ-C30 responses were scored according to the EORTC scoring manual and linearly transformed into standardized 0–100 scores. To ensure consistent interpretation of score direction across domains, differences for global health status and functional scales were calculated as baseline score minus postoperative score; therefore, positive values indicate postoperative worsening and negative values indicate postoperative improvement relative to baseline. For symptom scales, differences were calculated as postoperative score minus baseline score; therefore, positive values indicate greater postoperative symptom burden relative to baseline. Overall time effects were evaluated using the Friedman test. Clinically relevant post hoc pairwise comparisons are presented separately in Table 4. The magnitude of score changes should be interpreted cautiously, as statistical significance does not necessarily indicate clinically meaningful change. Functional/global and symptom domains were presented in separate visual sections to improve interpretability. * *p* < 0.05 indicates statistical significance.

**Table 5:** Postoperative morbidity profile.

Severity	n (%)
Minor complications (Clavien–Dindo I–II)	106 (73.6)
Major complications (Clavien–Dindo III–IV)	38 (26.4)
Reoperation	13 (9.0)
Anastomotic leakage	5 (3.5)
Chylous fistula	11 (7.6)
Intestinal fistula	4 (2.8)
Pancreatic fistula	9 (6.3)
Bile fistula	1 (0.7)
30-day mortality	1 (0.7)
90-day mortality	4 (2.8)

Abbreviations: Clavien–Dindo classification. Note: Minor complications were defined as Clavien–Dindo grade I–II, and major complications as grade III–IV. To improve the clinical interpretability of postoperative morbidity, reoperation, selected complication-specific events, and time-based mortality outcomes were also reported separately. Thirty-day and 90-day mortality were calculated according to the interval between the operation date and the recorded death date.

**Table 6:** Exploratory subgroup summary of selected QoL domains according to postoperative complication severity.

Domain	Time Point	Minor Complications	Major Complications
Global health status	Pre-op	96.6 ± 7.1	96.1 ± 7.2
	Post-op day 14	59.2 ± 14.5	50.7 ± 20.2
	Post-op 1 month	58.0 ± 15.4	50.0 ± 21.1
	Post-op 12 months	68.4 ± 27.9	74.2 ± 22.5
Physical functioning	Pre-op	100.0 ± 0.0	99.3 ± 5.5
	Post-op day 14	70.6 ± 19.6	60.9 ± 32.0
	Post-op 1 month	71.6 ± 21.4	61.4 ± 32.2
	Post-op 12 months	80.1 ± 34.6	84.8 ± 26.1
Social functioning	Pre-op	100.0 ± 0.0	99.6 ± 2.7
	Post-op day 14	73.7 ± 20.2	61.8 ± 35.5
	Post-op 1 month	73.1 ± 21.0	61.4 ± 37.6
	Post-op 12 months	79.3 ± 35.9	83.9 ± 29.2
Pain *	Pre-op	0.0 ± 0.0	1.8 ± 10.8
	Post-op day 14	42.3 ± 22.1	54.4 ± 31.6
	Post-op 1 month	41.5 ± 22.3	54.8 ± 31.9
	Post-op 12 months	24.7 ± 39.4	24.5 ± 34.9
Fatigue *	Pre-op	0.0 ± 1.5	1.5 ± 9.0
	Post-op day 14	35.6 ± 22.2	49.1 ± 32.2
	Post-op 1 month	34.3 ± 25.2	49.4 ± 35.1
	Post-op 12 months	22.7 ± 38.3	20.5 ± 31.9

Abbreviations: QoL, quality of life. * For symptom scales, higher scores indicate greater symptom burden. Note: Values are presented as mean ± SD after transformation to 0–100 EORTC scores. For global health status and functional scales, higher scores indicate better QoL/functioning. Baseline values should be interpreted in light of the selected operable nature of the cohort. These subgroup values are presented descriptively and were not evaluated using adjusted between-group longitudinal modeling.

**Table 7:** Exploratory subgroup summary of selected QoL domains according to primary tumor group.

Domain	Time Point	Colorectal	Gastric	Other *
Global health status	Pre-op	95.5 ± 7.9	95.6 ± 7.5	98.3 ± 5.1
	Post-op day 14	59.9 ± 15.3	49.1 ± 19.6	55.4 ± 16.5
	Post-op 1 month	58.4 ± 15.6	46.1 ± 22.3	56.0 ± 16.3
	Post-op 12 months	67.0 ± 31.0	73.1 ± 17.4	73.1 ± 21.5
Physical functioning	Pre-op	99.6 ± 3.8	100.0 ± 0.0	100.0 ± 0.0
	Post-op day 14	67.8 ± 24.3	59.3 ± 29.2	71.8 ± 20.0
	Post-op 1 month	67.8 ± 26.0	59.6 ± 29.4	74.1 ± 20.4
	Post-op 12 months	76.1 ± 36.9	88.2 ± 20.4	86.8 ± 27.7
Social functioning	Pre-op	99.8 ± 1.9	100.0 ± 0.0	100.0 ± 0.0
	Post-op day 14	72.8 ± 25.2	54.4 ± 32.3	73.5 ± 20.9
	Post-op 1 month	71.3 ± 27.4	54.4 ± 32.3	74.1 ± 21.3
	Post-op 12 months	76.9 ± 37.8	76.9 ± 34.4	86.5 ± 28.3
Pain †	Pre-op	0.9 ± 7.6	0.0 ± 0.0	0.0 ± 0.0
	Post-op day 14	43.6 ± 23.9	55.3 ± 31.5	44.6 ± 24.9
	Post-op 1 month	44.1 ± 25.0	55.3 ± 31.5	42.5 ± 24.1
	Post-op 12 months	29.0 ± 42.1	28.2 ± 32.9	17.7 ± 32.7
Fatigue †	Pre-op	0.7 ± 6.4	0.0 ± 0.0	0.0 ± 0.0
	Post-op day 14	38.0 ± 25.7	47.4 ± 32.0	37.9 ± 23.0
	Post-op 1 month	37.7 ± 29.2	48.5 ± 31.9	35.4 ± 26.3
	Post-op 12 months	25.9 ± 40.1	23.9 ± 35.7	16.7 ± 31.2

QoL, quality of life; Values are presented as mean ± SD after transformation to 0–100 EORTC scores. For global health status and functional scales, higher scores indicate better quality of life/functioning. † For symptom scales, higher scores indicate greater symptom burden. Baseline values should be interpreted in light of the selected operable nature of the cohort. These subgroup values are presented descriptively and were not evaluated using adjusted between-group longitudinal modeling. Other * primaries included ovarian cancer, pseudomyxoma peritonei, and malignant mesothelioma.

**Table 8:** Exploratory subgroup summary of selected QoL domains according to ostomy status.

Domain	Time Point	No Ostomy	Any Ostomy
Global health status	Pre-op	97.4 ± 6.0	94.4 ± 8.6
	Post-op day 14	57.4 ± 16.5	56.0 ± 16.9
	Post-op 1 month	56.1 ± 17.0	55.6 ± 17.7
	Post-op 3 months	64.0 ± 19.4	61.0 ± 19.0
	Post-op 6 months	69.5 ± 20.3	67.7 ± 22.6
	Post-op 12 months	72.3 ± 23.8	64.7 ± 31.7
	Post-op 18 months	77.5 ± 26.2	69.4 ± 32.7
	Post-op 24 months	81.9 ± 26.5	75.0 ± 33.9
Physical functioning	Pre-op	99.7 ± 3.4	100.0 ± 0.0
	Post-op day 14	69.7 ± 21.6	64.6 ± 28.0
	Post-op 1 month	71.1 ± 22.0	64.2 ± 30.2
	Post-op 3 months	83.9 ± 23.6	73.3 ± 31.1
	Post-op 6 months	87.7 ± 23.1	80.6 ± 28.9
	Post-op 12 months	86.6 ± 25.9	69.8 ± 41.8
	Post-op 18 months	86.7 ± 26.0	77.2 ± 38.2
	Post-op 24 months	87.7 ± 28.7	78.4 ± 39.3
Social functioning	Pre-op	99.8 ± 1.7	100.0 ± 0.0
	Post-op day 14	71.1 ± 25.0	69.6 ± 26.8
	Post-op 1 month	70.4 ± 25.6	69.2 ± 29.4
	Post-op 3 months	81.6 ± 27.0	76.0 ± 32.0
	Post-op 6 months	85.6 ± 25.7	82.9 ± 28.3
	Post-op 12 months	83.9 ± 30.6	73.0 ± 40.5
	Post-op 18 months	84.6 ± 31.0	81.0 ± 33.8
	Post-op 24 months	87.8 ± 28.5	80.9 ± 37.0
Pain *	Pre-op	0.7 ± 6.7	0.0 ± 0.0
	Post-op day 14	44.9 ± 25.4	46.7 ± 25.7
	Post-op 1 month	44.0 ± 24.8	47.1 ± 28.0
	Post-op 3 months	27.4 ± 29.5	33.3 ± 34.7
	Post-op 6 months	19.8 ± 29.2	25.2 ± 34.0
	Post-op 12 months	20.2 ± 33.5	34.5 ± 45.4
	Post-op 18 months	19.5 ± 34.0	25.0 ± 39.3
	Post-op 24 months	16.4 ± 33.6	25.0 ± 42.5
Fatigue *	Pre-op	0.6 ± 5.6	0.0 ± 0.0
	Post-op day 14	37.9 ± 25.6	42.0 ± 26.2
	Post-op 1 month	37.9 ± 28.3	39.4 ± 30.0
	Post-op 3 months	23.5 ± 29.8	29.7 ± 34.7
	Post-op 6 months	18.8 ± 28.7	22.7 ± 32.2
	Post-op 12 months	17.5 ± 32.0	32.5 ± 43.5
	Post-op 18 months	19.0 ± 33.2	23.5 ± 37.3
	Post-op 24 months	15.4 ± 32.2	23.9 ± 41.0

Abbreviations: QoL, quality of life. * For symptom scales, higher scores indicate greater symptom burden. Note: Values are presented as mean ± SD after transformation to 0–100 EORTC scores. For global health status and functional scales, higher scores indicate better QoL/functioning. “Any ostomy” included ileostomy and colostomy. These subgroup values are presented descriptively and were not evaluated using adjusted between-group longitudinal modeling.

## Data Availability

The data presented in this study are available on request from the corresponding author. The data are not publicly available because they were used under license for the current study.

## Abbreviations

The following abbreviations are used in this manuscript:

ASA	American Society of Anesthesiologists
CC-0	Complete cytoreduction (no visible residual disease)
CI	Confidence interval
CRS	Cytoreductive surgery
EORTC	European Organisation for Research and Treatment of Cancer
ERAS	Enhanced recovery after surgery
HIPEC	Hyperthermic intraperitoneal chemotherapy
ICU	Intensive care unit
IQR	Interquartile range
NA	Not applicable
PC	Peritoneal carcinomatosis
PCI	Peritoneal Cancer Index
PRO	Patient-reported outcome
QoL	Quality of life
QLQ-C30	Core Quality of Life Questionnaire
QLQ-CR29	Colorectal Quality of Life Questionnaire
SD	Standard deviation
